# Substantial Vitamin D Supplementation Is Required during the Prenatal Period to Improve Birth Outcomes

**DOI:** 10.3390/nu14040899

**Published:** 2022-02-21

**Authors:** Bruce W. Hollis, Carol L. Wagner

**Affiliations:** 1 Division of Neonatology, Shawn Jenkins Children’s Hospital, Medical University of South Carolina, Charleston, SC 29425, USA; wagnercl@musc.edu; 2 Darby Children’s Research Institute, Medical University of South Carolina, Charleston, SC 29425, USA

## 1. Introduction

Vitamin D supplementation during pregnancy has been studied since the early 1980′s and, while many clinical trials have been performed, we remain at a crossroads in our conclusions about vitamin D’s effects during pregnancy and the optimal dose and timing of supplementation. The importance of vitamin D during pregnancy surrounds its immunological functions rather than its endocrine role in the regulation of calcium, although that role is certainly not to be minimized. 

Initial findings from vitamin D research showed that vitamin D was required to normalize calcium homeostasis. We are not saying that this finding was not of utmost importance; rather, what we are saying is that this initial finding “blinded” researchers for decades with respect to the other essential intracrine and paracrine functions of vitamin D, including cancer prevention and treatment, immune regulation, as well as many other skeletal functions [1]. On the very top of this list would be vitamin D’s ability to greatly improve birth outcomes. This claim is largely based on research conducted in our laboratory [2,3,4,5,6].

What is vastly underappreciated about vitamin D during pregnancy is the unrivaled metabolic changes of the vitamin that take place, and which remain unexplained. First, massive amounts of 1,25(OH)_2_D begin to be produced and released into the maternal circulation as soon as placental implantation occurs [2]. The concentrations of 1,25(OH)_2_D circulating during pregnancy are superphysiologic, sometimes reaching 300 pg/mL [2]. This amount of circulating 1,25(OH)_2_D in a nonpregnant human would result in potentially fatal hypercalcemia. In our own experience, hypercalcemia due to excessive circulating 1,25(OH)_2_D in conditions outside of pregnancy can occur as levels reach only 80 pg/mL.

During pregnancy, where is this excessive amount of 1,25(OH)_2_D being produced? There are three possibilities: maternal kidney, fetal kidney, or placenta. All three organs can produce 1,25(OH)_2_D; however, it is unlikely coming from fetal kidneys or placenta, especially early on following conception, as fetal kidneys do not yet exist, and the placenta is miniscule with only layers of cells growing out from the blastocyst to form connections with the expanding lining of the uterus beginning around week 4-to-5 of gestation. Further, an earlier in vivo study points to maternal kidney production [7]. In this study, a woman became pregnant who did not have functioning kidneys to produce 1,25(OH)_2_D and was thus given 100,000 IU/day of vitamin D to maintain a normal circulating calcium levels [7]. During her pregnancy, she failed to elevate her circulating 1,25(OH)_2_D concentration, even though she had a normal functioning placenta, and her fetus had normally functioning kidneys (as evidenced by a normal amniotic fluid volume throughout pregnancy and normal kidney function after delivery). What was not functioning normally was her renal Cyp27b1, and thus, she could not produce or increase her circulating level of 1,25(OH)_2_D [7]. It is likely that this woman’s circulating 25(OH)D concentrations were at least 500 ng/mL and were being substituted for 1,25(OH)_2_D in cellular processes, thus affording her better health and immune protection.

## 2. Fundamental Questions

Two key questions came from this research. First, how does the maternal renal Cyp27b1 enzyme, which, under normal conditions, is under extraordinary feedback metabolic control, “lose that control” during pregnancy and produce “toxic” amounts of circulating 1,25(OH)_2_D? Second, how is it possible that these massive circulating 1,25(OH)_2_D levels are tolerated during pregnancy without incurring fatal hypercalcemia in either the mother or fetus, which would certainly occur outside of pregnancy? These questions remain to be answered.

The amount of vitamin D supplemented during pregnancy has long been a contentious subject due to the false association of vitamin D being a teratogen leading to Williams Syndrome [8,9]. This association, which is now known to be false, has caused generations of obstetricians and healthcare providers to fear vitamin D. Several studies have now clearly demonstrated vitamin D is safe at doses up to and exceeding 4400 IU/day [10,11,12,13,14,15,16,17,18,19,20,21,22,23]. In all of these studies, NOT A SINGLE ADVERSE EVENT has been associated with vitamin D supplementation [10,11,12,13,14,15,16,17,18,19,20,21,22,23]. Thus, it is now known that this dose of vitamin D can be safely administered during pregnancy. In fact, this level of supplementation is required during pregnancy to maintain a circulating level of 25(OH)D at a minimum of 40 ng/mL, which is required to support the optimal production of 1,25(OH)_2_D during pregnancy [2]. The question, from this data, remains: what would be the advantage of achieving this threshold?

A lack of vitamin D is long known to have adverse effects on skeletal development [10]. In fact, we used vitamin D and its ability to improve calcium homeostasis as the primary specific aim to obtain funding from the National Institutes of Health (NIH) to conduct a clinical trial in 2002 [2]. Ultimately, this trial was funded to study the administration of up to 4000 IU/day during pregnancy, starting at 12–16 weeks of gestation. Our primary goal was to determine how much vitamin D was required to achieve an adequate vitamin D status as defined by circulating 25(OH)D without compromising safety. It should be mentioned that this trial was so controversial that we had to obtain approval from the Food and Drug Administration (FDA) in the form of an investigational new drug (IND) application and number (#66,346), and the trial was conducted under their supervision. What this multiyear study ultimately demonstrated was that vitamin D supplementation during pregnancy could reduce birth complications. This benefit was not a primary endpoint of our original study because, in 2002, we did not know enough to even ask that question.

## 3. Pregnancy Outcomes through the Lens of Clinical Trials

When our study was completed and the results were presented at a Vitamin D Workshop in Brugge, Belgium in 2009, to put it simply, the data were not believed, nor were the positive effects of vitamin D on birth outcomes and complications viewed as plausible. With much effort, a few years later, our study was published in two parts [2,3]. These results spurred a large field of investigation that resulted in several additional studies [13,14,15,19,20,21,23,24]. Many of these clinical trials have validated the positive effects of prenatal vitamin D on birth outcomes [13,14,15,19,20,21,24] while a few have not [23]. However, as mentioned previously, all have shown vitamin D supplementation up to 4400 IU/day during pregnancy to be safe [10,11,12,13,14,15,16,17,18,19,20,21,22,23,24].

The study by Roth et al. published in 2019 highlights how NOT to reproduce past positive studies [23]. To do that, a study unlike the previous positive studies must be designed, supplementing far too little vitamin D at a timepoint far too late in gestation to influence the conditions that are being monitored, such as maternal safety and fetal growth monitoring [23]. It is likely that the intent was to see if later vitamin D supplementation could influence pregnancy outcomes, but it failed to consider vitamin D’s demonstrated effect on placentation and early pregnancy. It was not surprising that the results were negative in demonstrating a health benefit of vitamin D dosed in this manner. Post-publication letters to the editor of the *New England Journal of Medicine (NEJM)* were submitted and responses published [25,26]. These exact issues were raised in the letters and serve as a reminder of what must be considered when designing nutrient studies.

Recent studies have demonstrated that vitamin D needs to be administered as early in gestation as possible [24,27,28]. In fact, it appears to be critical to provide vitamin D in the preconception period to have the maximum protective effect with respect to preeclampsia [24,27]. This is likely because vitamin D is essential in the first trimester of pregnancy to ensure proper placental as well as lung development to prevent birth complications and childhood asthma. Anyone still doubting the substantial genomic effect imparted by vitamin D should review the following publications [24,28,29]. Specifically, the effect of vitamin D on immune regulation is truly astounding [24]. Providing vitamin D after this critical developmental period appears not to correct these developmental deficiencies, and thus, the failure of poorly designed clinical trials to show benefit from vitamin D supplementation [23]. A significant limitation in all of this is that preconception studies have only been observational. Ideally, preconception trials need to be randomized controlled trials; however, this is unlikely to happen because of the expense such a study would incur.

## 4. The Issues with Randomized Control Trials Applied to Nutrient Research

A side note about randomized controlled trials (RCTs) for nutrient research, especially vitamin D, is warranted. RCTs were designed for pharmaceutical trials, NEVER for nutrients. With respect to vitamin D when RCTs are employed, it is a miracle that any meaningful data are obtained. First, unlike pharmaceutical trials, everyone in a nutrient trial has some of that nutrient in their system so that fact alone represents a major confounding factor [29]. Secondly, non-compliance in studies is a major issue. Let us look at our 2011 study as an example [2]. During the study, we were blinded to treatment. Participants who failed to return pills or who had fewer pills taken during each monthly visit could not be removed because that would violate the intent-to-treat model. In *post hoc* analysis, comparing per protocol to intention-to-treat analyses, we found significant variability in certain women’s circulating 25(OH)D concentrations that aligned with the pill count data. When pregnant participants in the 4000 IU/day supplementation group failed to increase their circulating 25(OH)D concentration, and taken with the pill count data, it appeared likely that those participants were not being adherent to the daily dosing regimen [30]. In the intention-to-treat analyses, these non-compliant participants were analyzed in their assigned group, compromising the study in favor of null results, which has happened in several of our studies [2,5,14,21,30]. One way to overcome this is to use a population where all participants are deficient in vitamin D at the study entrance, which results in notable, significant positive pregnancy and birth outcomes [20,22].

The challenges of nutrient clinical trials are highlighted in these examples, when the failure of certain randomized clinical trials to demonstrate the benefits when dosing is suboptimal, or when nutrient research constraints are not applied. The next steps are to integrate the basic science findings as the center piece of nutrient clinical studies that are expansive rather than reductionistic and to design clinical trials that encompass nutrient physiology. Such an approach will further our understanding of the role that vitamin D plays during pregnancy to ultimately enhance pregnancy outcomes.

## 5. Limitations of Meta-Analyses in Nutrient Research

As far as meta-analyses of vitamin D and pregnancy studies are concerned, lumping in together studies performed with insufficient achieved levels of total circulating 25(OH)D and/or late gestational initiation of supplementation should be viewed with skepticism. Indeed, a recent meta-analysis on this topic omitted data from our seminal 2011 publication [31]. This paper misrepresented what we reported from our study by not citing the follow-up analysis from that study [3], where it was stated that there was no protection with respect to birth complications when, in fact, there was [3]. Further, that meta-analysis failed to include what we consider to be the best pregnancy vitamin D supplementation RCT performed thus far, where there were markedly worse pregnancy complications in those who were deficient compared to those who were supplemented with higher vitamin D doses [22]. The results were considered so significant that they warranted an editorial published in the journal [32].

## 6. Safety Considerations with Vitamin D Supplementation during Pregnancy

We remain at a crossroads with respect to using vitamin D during pregnancy to improve the birth complication rate. Naysayers continue to ask for more studies based on safety concerns, even though not a single adverse event has been observed in these studies, many of which were performed under FDA supervision. The fact remains that vitamin D is the only substance associated with decreased preeclampsia rates and subsequent preterm birth [5,6,22,24,33]. If vitamin D was a pharmaceutical, it would be worth billions of dollars. In our opinion, this is likely a major factor in the nonacceptance of vitamin D for this purpose, where vitamin D, which is essentially free and without a sponsor, is a major competitor to other, less efficacious products sponsored by pharmaceutical companies.

At this time, we should not look for any guidance on vitamin D supplementation dosing from organizations such as the National Institute of Medicine (NIM), formally known as the Institute of Medicine, because their latest guidance was published more than 10 years ago before any of the new data referenced here were even published [34]. It is expected that the NIM will produce a revised report within the next five years and that this report will be more encompassing, taking into account both vitamin D’s endocrine function and its now well-established role as an effector of immune function.

## 7. Conclusions

In closing, we would like to quote Sir Robert Hutchinson, a physician who lived in the United Kingdom (1871–1960) and was an international leader in childhood nutrition and rickets [35]: “From the inability to let well alone; from too much zeal for the new and contempt for what is old; from putting knowledge before wisdom, science before art, and cleverness before common sense; from treating patients as cases, and from making the cure of the disease more grievous than the endurance of the same, Good Lord, deliver us.” His words are as relevant today in the 21st century as they were a century ago. Our only hope is that our “cleverness” does not outwit human endurance and our acceptance of vitamin D’s role in everyday health.
